# Effect of dry and wet finishing and polishing on color change and opacity of nanofill and nanohybrid composites

**DOI:** 10.1186/s12903-024-03944-0

**Published:** 2024-02-28

**Authors:** Mehdi Molaei, Anoosh Mohammadzadeh, Amir Ghasemi, Mehdi Badiee

**Affiliations:** 1 School of Dentistry, Shahid Beheshti University of Medical Sciences, Tehran, Iran; 2 Dental Research Center, Research Institute of Dental Sciences, Shahid Beheshti University of Medical Sciences, Tehran, Iran; 3 School of Dentistry, Guilan University of Medical Sciences, Rasht, Iran

**Keywords:** Composite resin, Color, Opacity, Nano particle

## Abstract

**Background:**

As superior esthetic is one of the main reasons for using composite resins, it is very important to be familiar with factors and techniques affecting their optical properties and appearance.

**Aim:**

The aim of this study was comparing the effect of finishing and polishing with and without water coolant, on the color change and opacity of composite resin materials.

**Methods:**

Composites used for preparing samples were Z250 (microhybrid), Z350XT (nanofilled), and Z550 (nanohybrid). Then divided into 4 groups of 5 depending on finishing and polishing technique (dry or wet) and time (immediate and after twenty-four hours). After polishing, samples were assessed using a spectrophotometer. Color change and opacity were determined. Data was analyzed using Kolmogorov-Smirnov, ANOVA and Tukey HSD tests.

**Results:**

Type of material at both time had a significant effect on ΔE and opacity. Our results in dry and wet technique immediately(T0) showed that the highest and lowest ΔE and opacity belong to Z350XT (*p* < 0.001). After Twenty-four hours (T24), opacity of Z250 in wet condition was higher than dry condition (*p* < 0.001).

**Conclusions:**

Wet or dry technique was only effective on color in immediate polishing. Regarding opacity, technique was only effective in case of delayed polishing.

## Background

Within the last decades, dental patients’ expectations toward esthetic restorations have increased incredibly. The reasons behind this trend referred to the inherent property of composite resins. The majority of patients desire that tooth-colored restorative materials used to be long lasting, withstand harsh oral environments and hence have high color stability [1, 2].

Finishing and polishing of resin composite is an important step to improve esthetics and longevity of these type of restorations [1–5]. The appearance of resin composite after finishing and polishing is related to type, morphology and size of their filler particles and the polishing methods and instruments as well [6–9]. Therefore, the finishing and polishing procedures are both affected by the technique and materials. In general, a smaller filler size with a higher filler loading volume contributes to better polishability and the ability to retain a smooth polish surface [10], thus giving a more aesthetically pleasing results. The wake of nanotechnology has seen the incorporation of nano-sized fillers into composite resins. Nano-filled composite resins with filler size of 5–100 nanometers, have been reported to have superior clinical performance and aesthetic qualities [11].

Because of the difficulty to assess the texture and luster of the restoration’s surface while finishing with water, some authors advocate dry finishing with an air spray [4]. Also, immediate finishing and polishing may cause plastic deformation because only 75% of the material is cured after 10 min [2]. Therefore, it has been suggested to delay finishing and polishing procedures until 24 h after light curing the material [12]. In the literature [13], it has been reported that increasing the heat produced during dry finishing and polishing application, may cause both disruption the filler/matrix bond and separation of the filler particles from the matrix. As a result, between the filler and matrix, the micro cracks or the interfacial gaps at the interface, allow stain penetration and may cause discoloration.

Although much work has been done to ascertain the color change of composites, but the timing, i.e. immediate or delayed finishing, and polishing under dry or wet conditions affecting the physical properties of the resins remain a controversial topic. In this light, the purpose of this study was to evaluate the effect of finishing and polishing time and condition on the color change and opacity of nanofilled, nanohybrid, and microhybrid composite restorative materials available in the market. The null hypothesis of this study is that dry finishing and polishing time may not affect the color and opacity of nanofilled, nanohybrid, and microhybrid composite restorative materials.

## Methods

In this in vitro study specimens were randomly divided in to groups according to the type resin-based composite [microhybrid composite (Filtek Z250 3 M ESPE, USA), nanohybrid composite (Filtek Z550, 3 M ESPE, USA) and nanofilled composite (Filtek Z350 XT, 3 M ESPE, USA)], the dry/wet of finishing and polishing procedure and the time of finishing and polishing procedures. The null hypothesis of this study is that dry finishing and polishing time may not affect the color and opacity of nanofilled, nanohybrid, and microhybrid composite restorative materials.

Twenty samples were fabricated of each composite resin using a stainless-steel mold (8 mm in diameter and 2 mm in thickness). Composites were applied to molds and placed between two transparent Mylar strips. A glass slab was also placed on top of the upper Mylar strip and a constant pressure was applied in order for the excess composite to leak out. Next, the samples were light-cured for 20 s according to the manufacturer’s instructions using a quartz tungsten halogen light curing unit (Optilux; Kerr Corporation, Middleton, WI, USA) with the intensity of 600 mW/cm^2^. By means of a radiometer, the output of the light was frequently monitored (Hilux, Benlioglu Dental). Immediately after curing, the samples were removed from the mold and were randomly divided into 4 groups as follows:

- Group 1: immediately wet finished and polished.
- Group 2: immediately dry finished and polished.
- Group 3: wet finished and polished after twenty-four hours.
- Group 4: dry finished and polished after twenty-four hours.

Medium to super-fine aluminum oxide disks (Optidisc, Kerr,USA) were used. The aluminum oxide disks were discarded after each use. Each disk was used in a circular motion applying light pressure for 20 s with a slow-speed hand piece (NSK Ti-Max electric hand piece, Japan). The revolutions per minute were set to 5000. To control the variability, one investigator, blinded to which material was being processed, performed all the finishing sand polishing procedures in a randomized order. All groups were stored in saline at 37 °C before analysis.

Before each series of measurements, the spectrophotometer (XRite CI64, Grand Rapids, MI, USA) was calibrated according to the manufacturer recommendations using the supplied white calibration standard. D65 illumination and 10° standard observation angle were selected. Three measurements were taken on a black background with the active point of the spectrophotometer in the center of each specimen. The average reading was subsequently used for data analysis. The color measurements were done before and after finishing according to groups immediately(T0) and after twenty-four hours (T24),. The color difference (Δ*E*) was calculated for each sample using the following equation: Δ*E* = [(Δ*L*)^2^ + (Δ*a*)^2^ + (Δ*b*)^2^] ½.

The opacity is defined as the ratio of the reflectance of a specimen disk when backed by a black standard to that when backed by a white standard. A black and a white cardboard were taken from X-Rite Color Checker Passport card.

SPSS software (version 21.0; IBM) was used for the statistical analysis. The level of significance was set at *P* = 0.05. The Kolmogorov- Smirnov test was used for checking for the normality of data distribution. Data were analyzed using Two-way and One-way ANOVA and Tukey’s HSD test.

## Results

Data are summarized in Table 1. At T0, type of materials and finishing condition affected ΔE (*P* < 0.001). In both wet and dry condition the lowest and highest ΔE was recorded for Z550 and Z350XT respectively at T0. Tukey HSD revealed that all materials significantly different in their ΔE. The highest ΔE in wet and dry condition was obtained with Z350XT at T0. All materials had significantly higher ΔE in dry condition in comparison with wet condition at T0 (*P* < 0.001). At T24, finishing condition neither did not affect ΔE (*P* = 0.829) nor type of materials. At T24 delayed finishing and polishing significantly affected ΔE in both wet and dry condition for Z350XT.

**Table 1:** Mean Δ*E* and opacity values and standard deviations for the tested materials and polishing procedures

Material	Time	Status	∆E ± SD	%Opacity ± SD
Z250	T_0_	Wet	5.388 ± 0.48	76.206 ± 1.02
		Dry	6.482 ± 0.15	76.558 ± 1.89
	T_24_	Wet	5.782 ± 0.50	78.546 ± 1.18
		Dry	5.202 ± 0.42	73.992 ± 2.18
Z350 XT	T_0_	Wet	5.868 ± 0.45	72.684 ± 1.47
		Dry	7.152 ± 0.46	73.800 ± 0.89
	T_24_	Wet	1.508 ± 0.23	72.614 ± 1.13
		Dry	1.350 ± 0.52	73.448 ± 1.12
Z550	T_0_	Wet	3.810 ± 0.35	78.966 ± 0.98
		Dry	4.986 ± 0.59	79.250 ± 1.90
	T_24_	Wet	3.488 ± 0.56	79.742 ± 1.22
		Dry	4.334 ± 0.37	78.612 ± 0.75

At T0, type of materials affected opacity (*P* < 0.001). But finishing condition did not affected opacity (*P* < 0.001). In both wet and dry condition the highest and lowest opacity was observed for Z550 and Z350XT respectively at T0. At T24 in wet condition Z550 showed significantly highest opacity values in all groups compared to other composite resins (*P* < 0.001). At T24, opacity of Z250 in wet condition was higher than dry condition (*P* < 0.001). Tukey HSD showed that at T24 there is no significant differences in opacity between wet and dry finishing condition for Z550 and Z350XT (*P* > 0.05).

## Discussion

Most dentists prefer to do the finishing and polishing step immediately after the light curing of the resin restoration, to improve marginal adaptation by closing the gap formed by polymerization shrinkage and finishing/polishing procedures [13]. Also it is more acceptable and cost effective for the patient and dentist. However, it is recommended to delay any finishing procedures until after hygroscopic expansion occurs to decrease the risk of fracture of the unsupported enamel surrounding the marginal gap [14]. Another reason for delayed polishing is that resin is only 75% cured after 10 min and immediate polishing may cause plastic deformation [13]. Due to such different points of view, we decided to compare immediate and delayed (after twenty-four hours) polishing in this study.

Optical properties of the dental composite resins were influenced by surface changes during restorative procedures of finishing and polishing [15]. In order to measure the color alterations on composite resin restorations objectively, some methods have been experienced. One of them is the spectrophotometry, which makes it possible to study several parameters related to color stability of composite resins [16]. The X-Rite Ci64 Portable Handheld Spectrophotometer measures precise sections of the visible light spectrum, within 400 to 700 nm, based on the reflection of specific body wavelengths, and translating them in values expressed in Δ*E* units [17]. This system have certain key components of light source, method of spectral separation or dispersion, and a detection system [18]. As we know reflected color of resin composites is affected by the background [19]. This has been quite a controversial topic in optical measurement. We preferred using the black background which is suitable for mimicking in vivo condition [20, 21].

The null hypothesis is rejected because our results showed that color changes at T0 were depends on type and wet/dry finishing and polishing condition which was in line with the findings of previous studies [15, 22, 23]. Shape of filler particles, their composition and distribution, percentage of filler particles, and type of resin can affect the color [15, 22]. In this study, our results at T0 showed that Δ*E* was higher following dry finishing and polishing compared to those subjected to wet finishing and polishing. It could be considered such a kind of stress as stated in the study of Bausch et al. However, increasing temperature stress above glass transition phase in dry finishing and polishing [24]. Moreover, in dry finishing and polishing, there is a risk of abrasive particles separation from the polishing tool that may embedded into composite surface and make a change in surface roughness [9]. As the surface roughness increase the color change may occur.

Our result showed that, in both wet and dry condition the highest ΔE was recorded for Z350XT at T0. This could be due to its sensitivity to polymerization procedure [25–27] because of a light-scattering effect in nano filled composites. These results are in accordance with those obtained by Kim et al. [25]., Lee et al. [26] and Rebeiro et al. [27] in which nano particles increased the light reflection which could be decreased degree of conversion [25, 27]. As to the color change after polymerization of resin composites, polymerization could cause a change of the refractive index of the matrix phase, making the materials less translucent due to the increased scattering [19]. These results are in accordance with the results of opacity at T0 and T24. In the present study, the lowest opacity reported for Z350XT. In line with the report of Kim et al. [25] When a particle shrinks to a fraction of the wavelength of visible light (400–800 nm), it will not scatter that particular light. In contrast, if the size of the particles is far below the wavelength of light, it will not scatter or absorb the light, resulting in the human eye’s inability to detect the particles. Thus, dental composites with tiny nanoparticles produce superior translucency and deliver optimal aesthetics [25].

Dry/ wet finishing and polishing did not affected opacity at T0 which is similar to Lee et al. Study [26]. In dry condition at T24, Z550 revealed the highest opacity which could be due to its composition. There are several factors that can influence the opacity and its opposite translucency of dental composite materials (the translucency of the component, the filler and matrix of the composite, the sizes of filler particle) [26]. Z550 as a nano hybrid composite contains a broad range of particle sizes(0.1–10 micron) for higher filler loading in comparison with Z250 as a micro hybrid composite(0.1-3 micron). The particle size discrepancies of the nano hybrid composite filler is able to cause the higher opacity of Z550 at T24 [25].

It has to be mentioned that the chemical composition, the different fillers types and sizes and the monomer quantities of resin composites are not often disclosed, in details, by the manufacturers.

Within the limitations, the authors aimed to mimic effects of the dry and wet finishing and polishing that may occur in the oral environment to estimate the clinical performance of resin composites. The optical properties of resin composites may be affected by the saliva, moisture, aging, and colorants from drinks and food in the oral environment over time. Additionally, mastication process can change the surface properties of resin composite and make them rough and discolored. It means that there are lots of factors in oral cavity that may accelerate the discoloration process. Further studies using different materials, formula and polishing methods should be conducted on resin composites. Operators should thus note that the optical properties of the resin composites may change in the long term. These results provide a foundation for further research and should be interpreted in the context of in vitro conditions. Clinical validation is required to fully understand the behavior of these materials in the oral cavity. Nonetheless, the study contributes to the existing body of knowledge and offers valuable insights for clinicians aiming to enhance the longevity and success of dental restorations.

## Conclusion

Color change of composites due to finishing and polishing process was always dependent on type of material. Wet or dry technique was only effective on Δ*E* in immediate finishing and polishing. Regarding opacity, technique was only effective in case of delayed polishing.

## Data Availability

The datasets used and/or analyzed during the current study are available from the corresponding author on reasonable request.
